# Supplementary material for: Endogenous aldehyde accumulation generates genotoxicity and exhaled biomarkers in esophageal adenocarcinoma
Source: Nat Commun. 2021 Mar 5;12:1454. doi: 10.1038/s41467-021-21800-5 (PMC7935981; doi:10.1038/s41467-021-21800-5)
Supplement: Supplementary file 1 — Supplementary Information [file 41467_2021_21800_MOESM1_ESM.pdf]

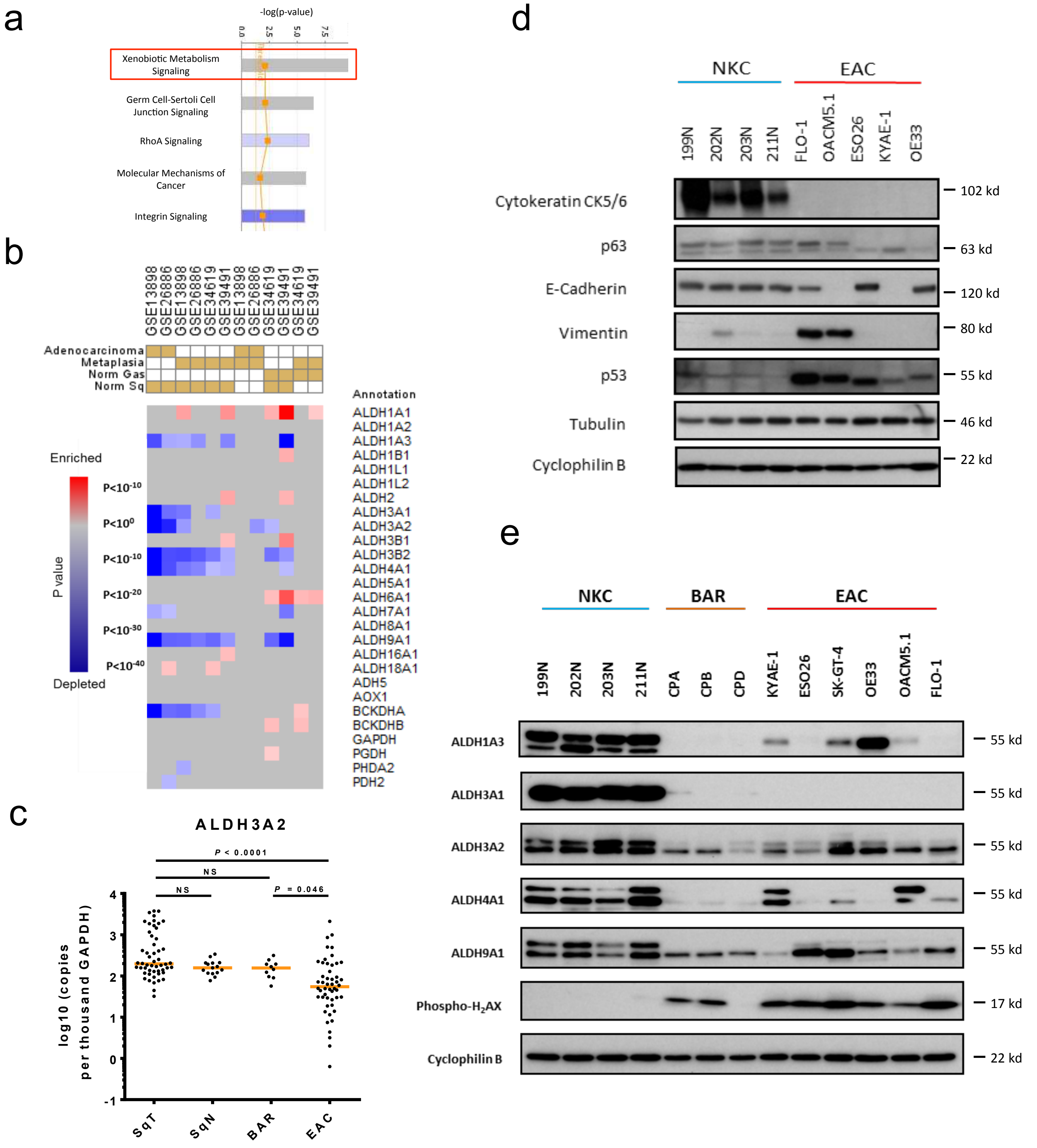

Supplementary Figure 1. Aldehyde detoxification loss is conspicuous in esophageal adenocarcinoma. a Ingenuity Pathway Analysis of GSE26886 SqN (*n* = 19) vs EAC (*n* = 21), core analysis using fold change >2 and *P* < 0.00001. b Heatmap of relative expression differences between the indicated tissue-types in the indicated studies, for the members of the 'Gene Ontology (v4.0) oxidoreductases acting on the OH of donors' geneset (P-values calculated with two-tailed Student's T-test, with Bonferroni-corrected significance threshold of 0.0000025. Blue indicates a negative fold change from squamous/metaplasia; red, positive fold change from squamous or metaplasia. Depth of color represents significance of difference). c *ALDH3A2* expression in the SqN and BAR tissues, compared to SqT and EAC data from Figure 1c. SqN, squamous sample from endoscopically normal oesophagus (*n* = 15 patients); SqT, cancer-adjacent histologically normal squamous sample (*n* = 67); BAR, Barrett's metaplasia (*n* = 10); EAC, esophageal adenocarcinoma (*n* = 67) d Western blot analysis of normal keratinocyte cultures (NKC) and esophageal cancer cell lines (EAC) for keratinocyte markers. e Western blot analysis of normal keratinocyte cultures, Barrett's cell lines and esophageal cancer cell lines for ALDH isoenzymes and phospho-H_2AX. Kruskall-Wallis test with Dunn's correction used in (c), **P* < 0.05, ***P* <0.01, ****P* < 0.001, *****P* < 0.0001. All blots are representative of three independent experiments with similar results. Source data are provided in the Source Data file.

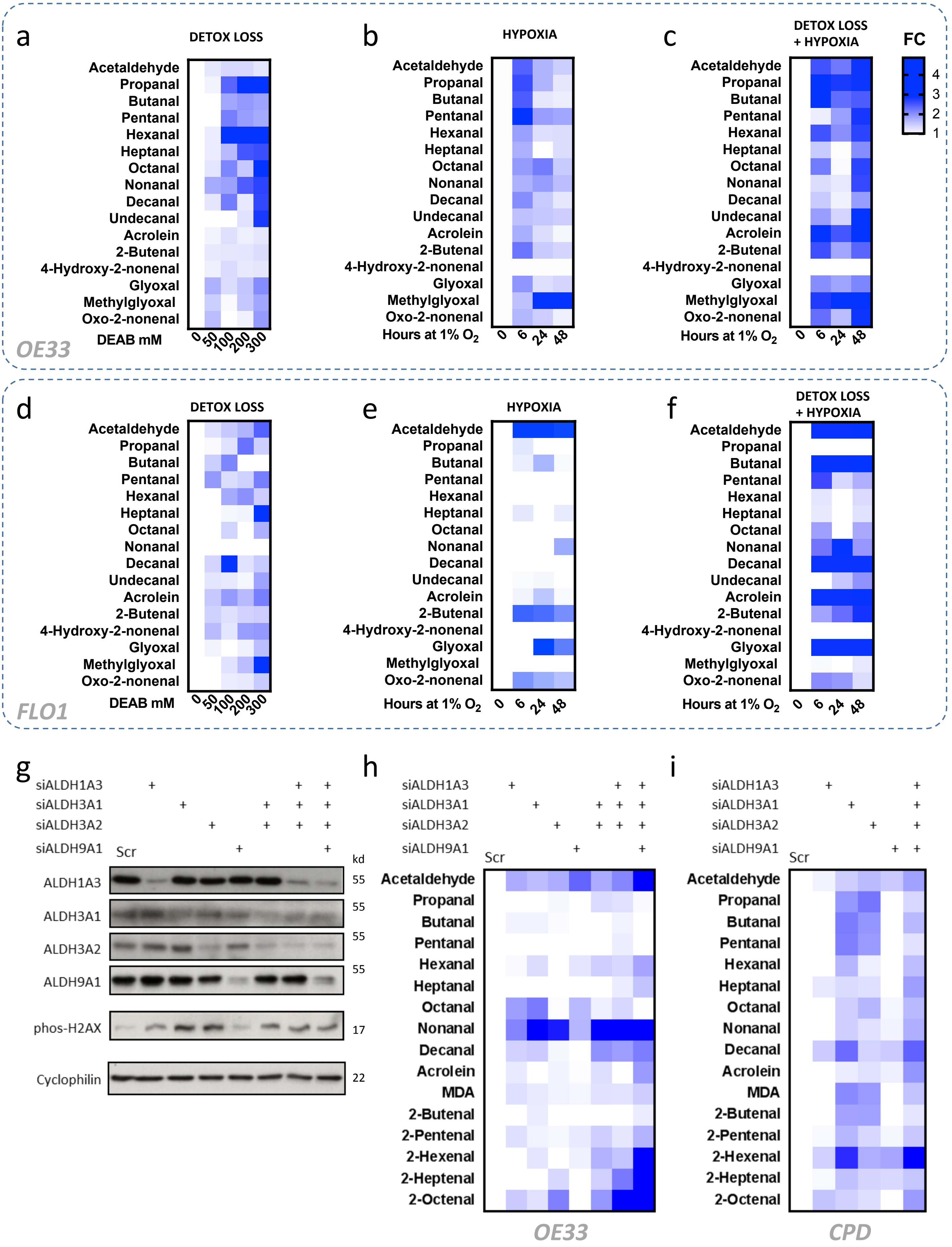

Supplementary Figure 2. Loss of aldehyde detoxification is sufficient to enrich endogenous aldehydes

Supplementary Figure 2. Loss of aldehyde detoxification is sufficient to enrich endogenous aldehydes a-f UPLC-MS/MS analysis of selected aldehydes in culture media. Heatmaps indicate fold change (FC) in normalized intensity from control. a,d Aldehyde fold-changes following incubation with diethylaminobenzaldehyde (DEAB). b,e Aldehyde fold-changes following incubation at 1% O_2. c,f Aldehyde fold-changes following incubation at 1% $O_{2,}$ in the presence of 200 mM DEAB. a-c OE33 cells used d-f FLO1cells used. g Western blot analysis of OE33 cells following transient transfection with siRNAs targeting the indicated ALDH isoforms h,i Aldehyde fold-changes following RNA interference of selected ALDH genes, in OE33 cells (h) and CPD cells (i). MDA, malondialdehyde; Scr, scrambled control RNA only. The siRNA immunoblot was repeated twice with the same result. Source data are provided in the Source Data file.

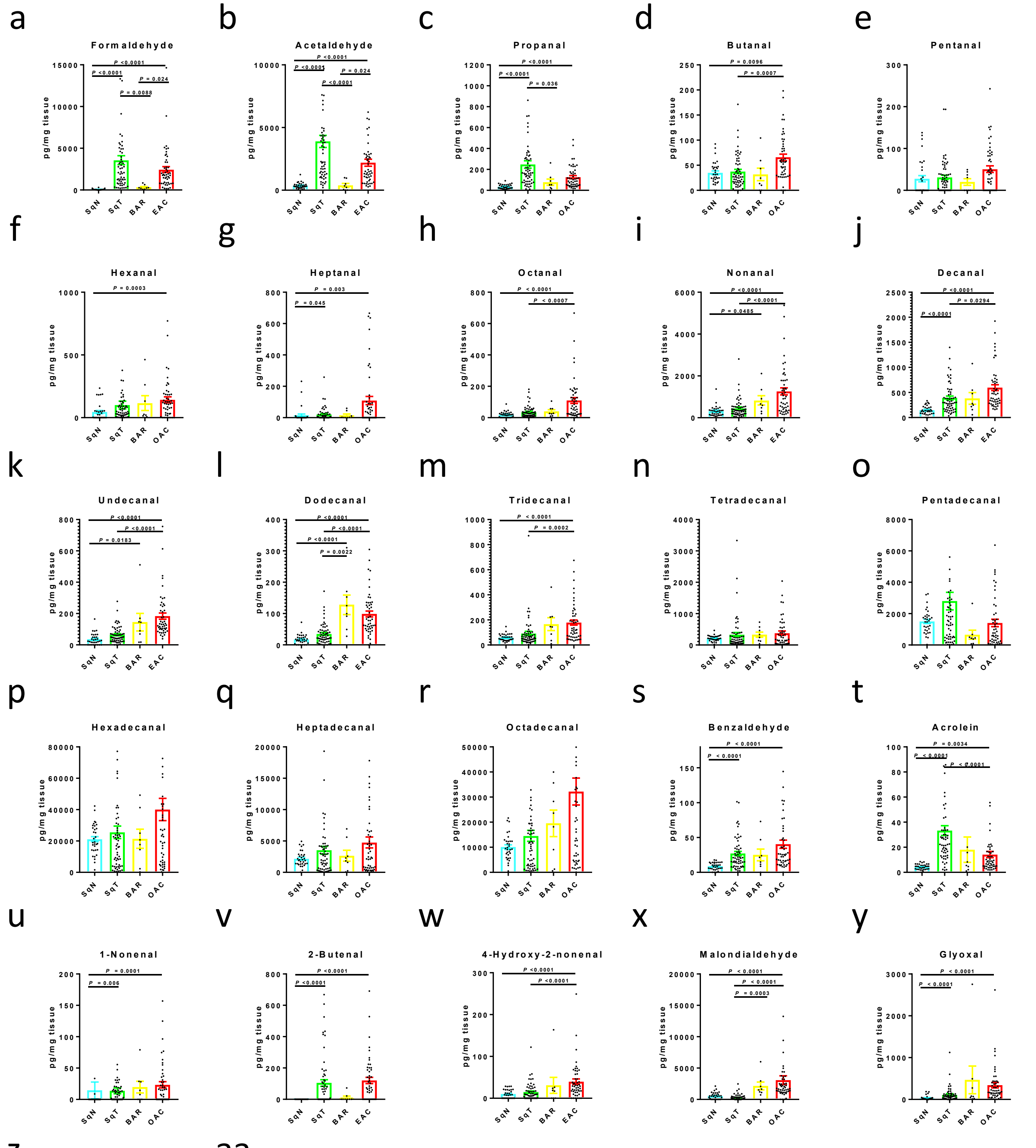

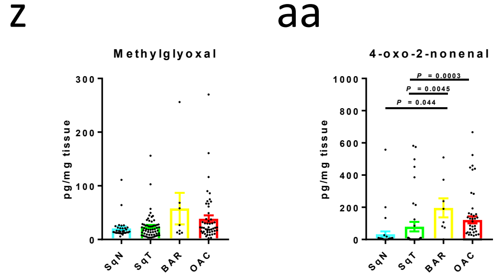

Supplementary Figure 3. Tissue aldehyde concentrations in esophageal endoscopic biopsies measured with UPLC-MS/MS. Squamous tissue from healthy patients (SqN, n = 32 independent patients), esophageal adenocarcinoma (EAC)-adjacent squamous tissue (SqT, n = 59), EAC-adjacent Barrett's (BAR, n = 8) and EAC (n = 59) were compared. HNE, 4-hydroxy-2-nonenal; SqN, squamous sample from endoscopically normal esophagus; SqT, EAC-adjacent squamous sample; BAR, Barrett's metaplasia; EAC, esophageal adenocarcinoma. Mean ± SEM provided. Kruskal-Wallis test with Dunn's correction. **P* < 0.05, ***P* <0.01, ****P* < 0.001, *****P* < 0.0001. Source data are provided in the Source Data file.

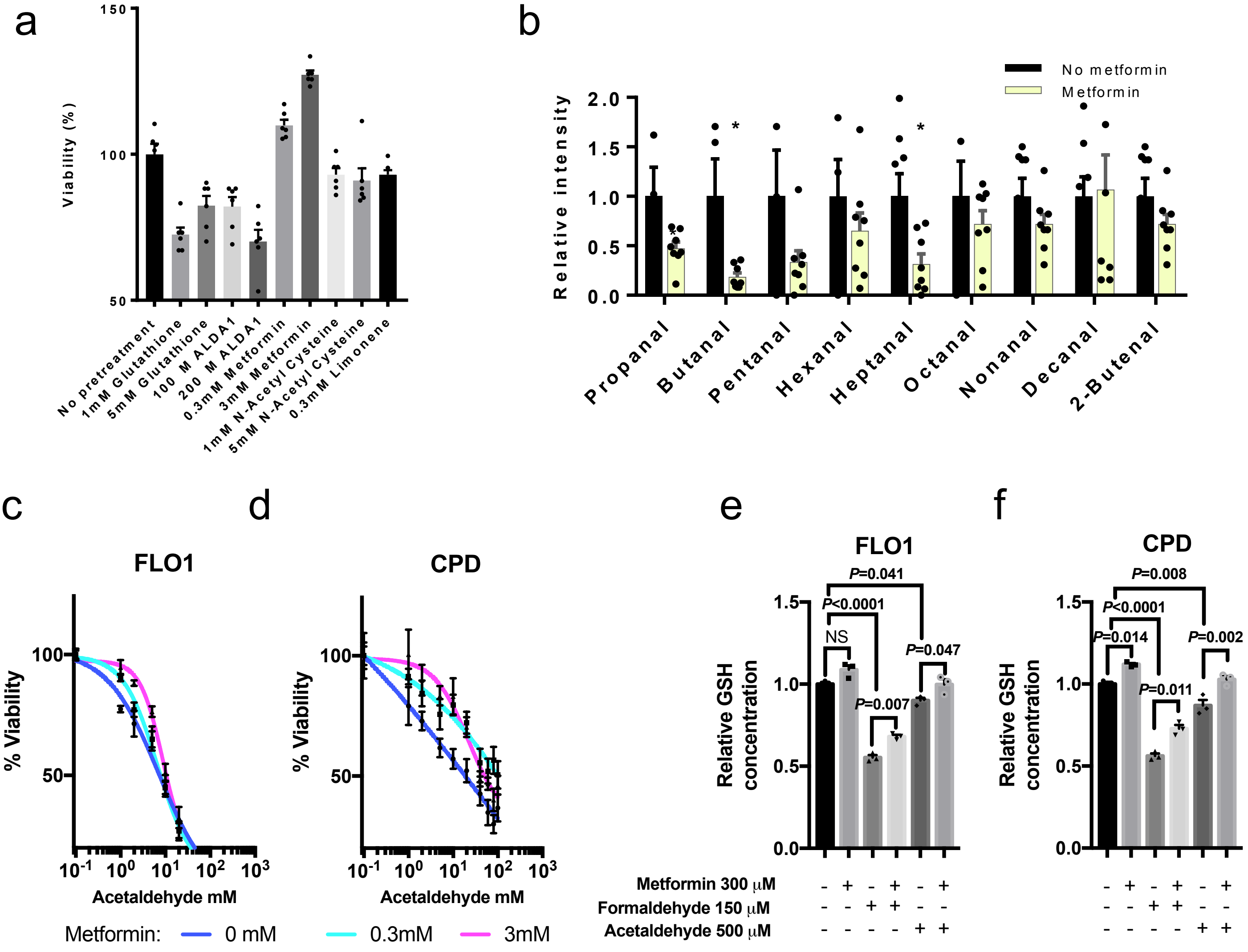

Supplementary Figure 4. Aldehydes cause EAC DNA damage, which can be reduced by metformin a Viability of FLO1 cells after 48 hours exposure to 1mM acetaldehyde, following pretreatment with the indicated anti-aldehyde therapeutics (n = 6 biologically independent samples) b Concentrations of exhaled aldehydes of EAC patients with diabetes, with and without metformin therapy. Breath data from Markar et al 2019, *n* = 16 (Butanal, *P* = 0.015; Heptanal, *P* = 0.028; two-tailed Mann-Whitney U-test). c,d Viability of FLO1 cells (c) or CPD cells (d) after 48 hours exposure to various concentrations of acetaldehyde, following pretreatment with the indicated concentrations of metformin (n = 6 biologically independent samples at each timepoint) e,f Ratio of reduced to oxidised glutathione in FLO1 cells (e) or CPD cells (f) pretreated with metformin and exposed to the indicated aldehydes for 5 hours (n = 3 biological replicates of technical triplicates). Mean ± SEM provided. Kruskall-Wallis with Dunn's correction or two-tailed Mann-Whitney U-test *$P < 0.05$, ***$P < 0.001$, ****$P < 0.0001$. Source data are provided in the Source Data file.

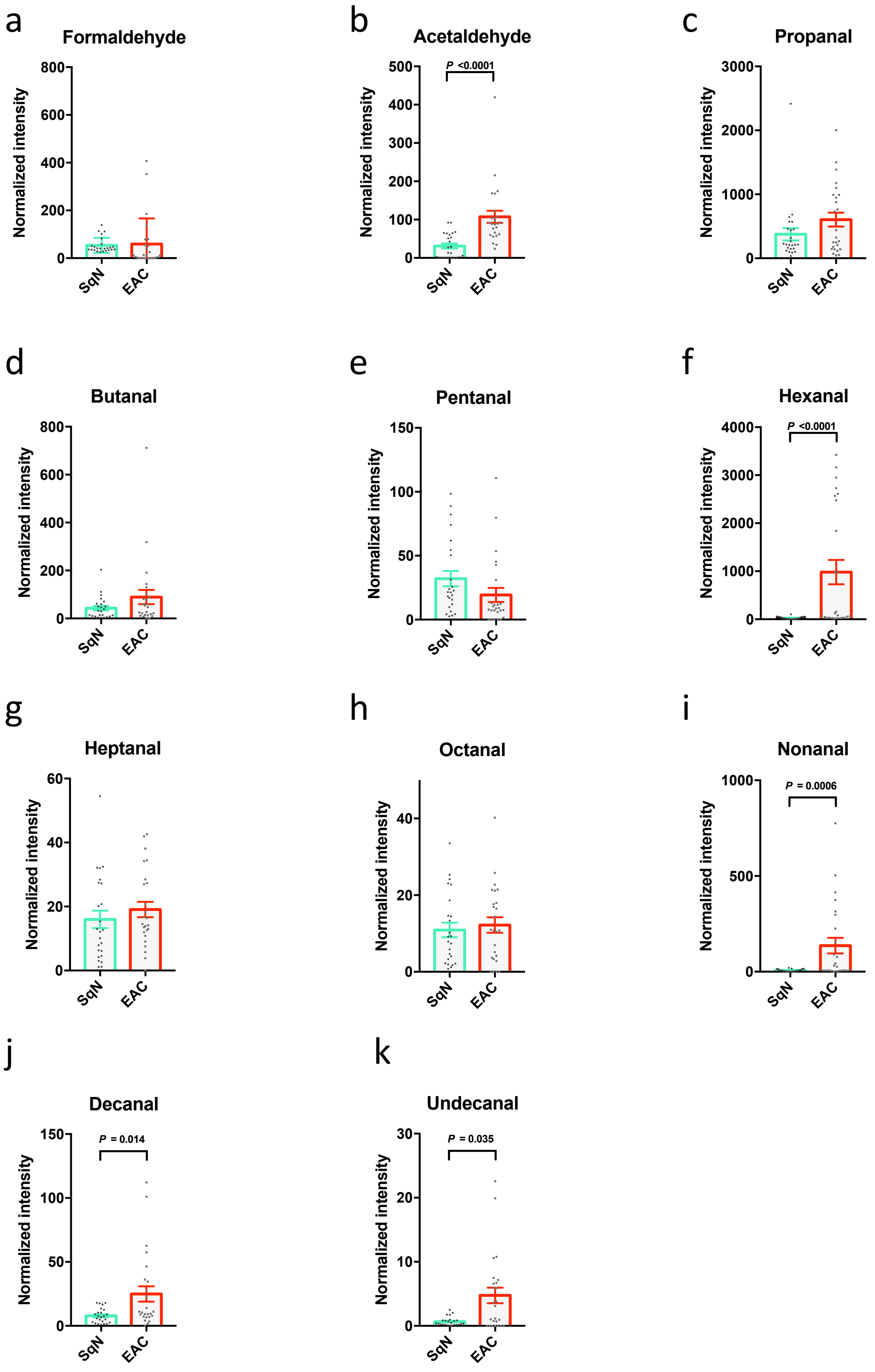

Supplementary Figure 5. Aldehyde concentrations in esophageal tissue headspace measured by PTR-TOF-MS. SqN, squamous sample from healthy volunteers (n = 24 independent volunteers); EAC, esophageal adenocarcinoma (n = 25 independent patients). Mean ± SEM provided. Two-tailed Mann-Whitney U-test *$P < 0.05$, **$P < 0.01$, ***$P < 0.001$, ****$P < 0.0001$. Source data are provided in the Source Data file.

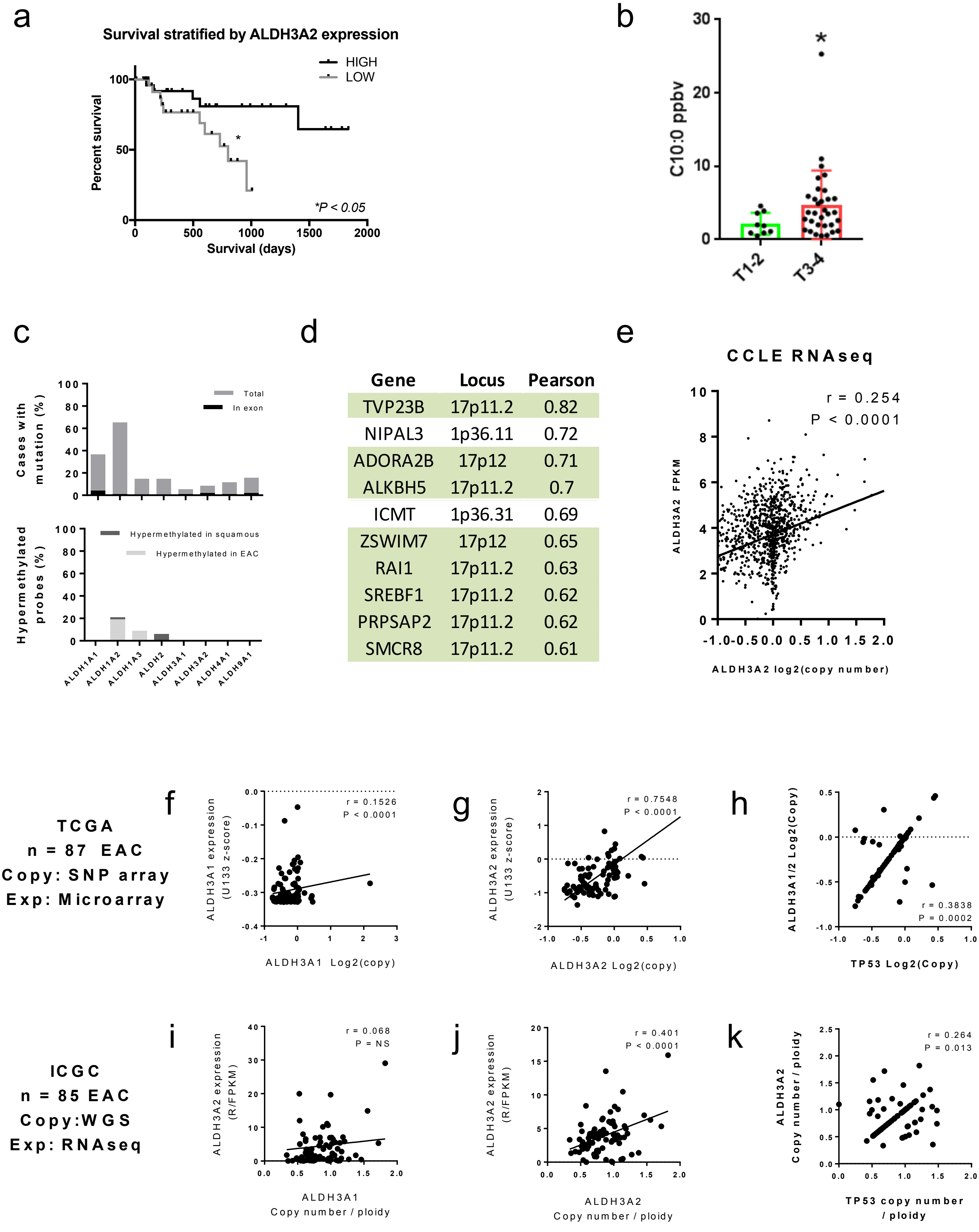

Supplementary Figure 6. Medium chain aldehydes link tissue metabolism to TP53 and prognosis

Supplementary Figure 6. Medium chain aldehydes link tissue metabolism to TP53 and prognosis. a EAC survival by *ALDH3A2* expression (n = 57 EAC cases with available survival data, data from The Cancer Genome Atlas, dichotomised to low and high expressing using the median FPKM value). Log-rank test, $P = 0.0202$. b Exhaled decanal concentrations by EAC tumor stage ($n = 41$). Two-tailed Mann-Whitney U-test, $P = 0.041$. Breath data from Markar et al 2018. c Mutation frequencies and methylation status in selected ALDH isoenzymes in EAC, TCGA data (n = 87). d Top 10 most correlated genes with *ALDH3A2* expression, TCGA RNA-seq fragments per kilobase per megabase (FPKM) expression data. Pearson correlation. e Correlation of *ALDH3A2* copy to expression (data from Cancer Cell Line Encyclopaedia data, n = 1094) two-tailed Pearson test. f-k Comparison of copy number to expression, or copy number to copy number using TCGA (f-h) or ICGC (i-k) data. Two-tailed Spearman's test. Mean ± SEM provided for technical triplicates of biological duplicates or triplicates, Two-tailed Mann-Whitney U-test or Kruskall-Wallis test, *P < 0.05, **P <0.01, ***P < 0.001, ****P < 0.0001. Source data are provided in the Source Data file.
