# Supplementary material for: Endogenous aldehyde accumulation generates genotoxicity and exhaled biomarkers in esophageal adenocarcinoma
Source: Nat Commun. 2021 Mar 5;12:1454. doi: 10.1038/s41467-021-21800-5 (PMC7935981; doi:10.1038/s41467-021-21800-5)
Supplement: Supplementary file 3 — Description of Additional Supplementary Files [file 41467_2021_21800_MOESM3_ESM.pdf]

Description of Additional Supplementary Files

File Name: Supplementary Data 1
Description: **Study materials and data sources**

File Name: Supplementary Data 2
Description: **Retention times and MS/MS settings for DNPH hydrazone quantification by UPLC-MS/MS**

File Name: Supplementary Data 3
Description: **Gene Set Enrichment Analysis results. P values calculated in the software using permutation test (P =). FDR = false discovery rate, FWER = forward-wise error rate**

File Name: Supplementary Data 4
Description: **Clinical parameters of participants in the ALDH3A1/2 immunoreactivity studies**

File Name: Supplementary Data 5
Description: **Clinical parameters of participants in the tissue aldehyde studies**

File Name: Supplementary Data 6
Description: **Cox proportional hazards model including ALDH3A1/2 immunoreactivity in the survival model.**

File Name: Supplementary Data 7
Description: **R code for exporting Targetlynx intensities**
