# Supplementary material for: Endogenous aldehyde accumulation generates genotoxicity and exhaled biomarkers in esophageal adenocarcinoma
Source: Nat Commun. 2021 Mar 5;12:1454. doi: 10.1038/s41467-021-21800-5 (PMC7935981; doi:10.1038/s41467-021-21800-5)
Supplement: Supplementary file 11 — Reporting Summary [file 41467_2021_21800_MOESM11_ESM.pdf]

nature research

Corresponding author(s): GB Hanna

Last updated by author(s): Dec 28, 2020

Reporting Summary

Nature Research wishes to improve the reproducibility of the work that we publish. This form provides structure for consistency and transparency in reporting. For further information on Nature Research policies, see our Editorial Policies and the Editorial Policy Checklist.

Statistics

For all statistical analyses, confirm that the following items are present in the figure legend, table legend, main text, or Methods section.

n/a	Confirmed	
☐	☒	The exact sample size (n) for each experimental group/condition, given as a discrete number and unit of measurement
☐	☒	A statement on whether measurements were taken from distinct samples or whether the same sample was measured repeatedly
☐	☒	The statistical test(s) used AND whether they are one- or two-sided<br>*Only common tests should be described solely by name; describe more complex techniques in the Methods section.*
☐	☒	A description of all covariates tested
☐	☒	A description of any assumptions or corrections, such as tests of normality and adjustment for multiple comparisons
☐	☒	A full description of the statistical parameters including central tendency (e.g. means) or other basic estimates (e.g. regression coefficient) AND variation (e.g. standard deviation) or associated estimates of uncertainty (e.g. confidence intervals)
☐	☒	For null hypothesis testing, the test statistic (e.g. F, t, r) with confidence intervals, effect sizes, degrees of freedom and P value noted<br>*Give P values as exact values whenever suitable.*
☒	☐	For Bayesian analysis, information on the choice of priors and Markov chain Monte Carlo settings
☒	☐	For hierarchical and complex designs, identification of the appropriate level for tests and full reporting of outcomes
☒	☐	Estimates of effect sizes (e.g. Cohen's d, Pearson's r), indicating how they were calculated

Our web collection on statistics for biologists contains articles on many of the points above.

Software and code

Policy information about availability of computer code

Data collection	LC-MS data was collected using Masslynx SCN909 (Waters Corporation). PTR-MS data was collected using PTR-MS viewer 3.2.2 (Iconicon). qPCR data was collected using SDS v2.4.1 for 7900HT (ThermoFisher). Immunoblot data was collected using manual film exposure. Immunohistochemistry data were collected with NanoZoomer 2.0HT (Hamamatsu). FISH images were collected using the Delta Vision Elite Imaging System (Imsol)
Data analysis	The following analysis software was used in this paper:<br>SNP calling and copy number analyses: ASCAT 2.3 (Crick Institute) & GATK 3.2.2 (Broad institute)<br>PTR-MS analysis: PTR-MS viewer 3.2.2.2 (Iconicon Analytik)<br>LC-MS data analysis: Targetlynx SCN855 (Waters Corporation)<br>Gene set enrichment analysis of expression datasets: GSEA ver 4.1 (Broad Institute) & Ingenuity Pathway Analysis 01-07 (Qiagen)<br>Automated image analysis for FISH studies: Cell Profiler (Broad Institute)<br>Metabolic data presentation and statistical testing: Metaboanalyst 4.0 (McGill) and GENE-E (Broad Institute)<br>General data presentation and statistical testing: RStudio version 1.1.456 (RStudio, Inc)<br>General data presentation and statistical testing: Prism 7 (GraphPad Software)<br>General data presentation and statistical testing: SPSS ver 26 (IBM)<br>Custom R code was developed to assist post-processing the UPLC-MS/MS data and is provided in Supplementary Table 7

For manuscripts utilizing custom algorithms or software that are central to the research but not yet described in published literature, software must be made available to editors and reviewers. We strongly encourage code deposition in a community repository (e.g. GitHub). See the Nature Research guidelines for submitting code & software for further information.

Data

Policy information about availability of data

All manuscripts must include a data availability statement. This statement should provide the following information, where applicable:

- Accession codes, unique identifiers, or web links for publicly available datasets
- A list of figures that have associated raw data
- A description of any restrictions on data availability

All experimental source data are provided with this paper in the source file. Legacy microarray expression studies re-analysed here (in Figure 1 and Supplementary Figure 1) are available at https://www.ncbi.nlm.nih.gov/gds, under the accession codes: GSE 26886 (https://www.ncbi.nlm.nih.gov/geo/query/acc.cgi?acc=GSE26886), GSE13898 (https://www.ncbi.nlm.nih.gov/geo/query/acc.cgi?acc=GSE13898), GSE39491 https://www.ncbi.nlm.nih.gov/geo/query/acc.cgi?acc=GSE39491) and GSE34619 (https://www.ncbi.nlm.nih.gov/geo/query/acc.cgi?acc=GSE34619). Data from The Cancer Genome Atlas (used in Figure 5 and Supplementary Figure 6) is available at: https://portal.gdc.cancer.gov/projects/TCGA-ESCA. Data from the International Cancer Genome Consortium (used in Supplementary Figure 6) is available at: https://ega-archive.org/studies/EGAS00001000725. Data from The Cancer Cell Line Encyclopedia (used in Supplementary Figure 6) is available at: https://portals.broadinstitute.org/ccle.

Field-specific reporting

Please select the one below that is the best fit for your research. If you are not sure, read the appropriate sections before making your selection.

☒ Life sciences ☐ Behavioural & social sciences ☐ Ecological, evolutionary & environmental sciences

For a reference copy of the document with all sections, see nature.com/documents/nr-reporting-summary-flat.pdf

Life sciences study design

All studies must disclose on these points even when the disclosure is negative.

Sample size

For in vivo ALDH expression analyses, a sample size was determined from GSE26886 expression distributions, taking $\alpha = 0.05$ and $\beta = 0.8$, which returned 5 (ALDH4A1) to 67 (ALDH1A1) paired samples (see Methods; thus, the sample size for the group overall was 67 tumour-normal pairs).

For the UPLC-MS/MS analysis of tissue aldehyde concentrations, a sample size for tumour-normal pairs was calculated following an initial pilot run of 10 tumour-normal pairs (not included in the published analysis). This returned a sample size of 59 to detect a 30% difference in decanal concentration, which included a 10% attrition rate for inadequate material.

Otherwise, statistical methods were not used to predetermine sample size. Number of sample was determined based on experimental approach, availability, feasibility required to obtain convincing results. Sample size was chosen based on our prior studies and published literature using the same types of assays to ensure statistically meaningful results. All studies were performed with at least three biologically independent samples per group.

Data exclusions

For all survival analyses in Figure 5, patients who died with 90 days of surgery were excluded from the analysis (predetermined exclusion) as the causes of death were surgical rather than cancer-specific. Otherwise, no data was excluded.

Replication

The ALDH mRNA expression studies were carried out in technical triplicate. The Immunophenotyping experiments were carried out in three biological replicates per patient (three different cores), read by two observors, and averaged. The tissue aldehyde LC-MS study was carried out 2-3 biological replicates depending on tissue availability per patient. All cell line immunoblots were representative images of three separate experiments. All colorimetric assays were representative of three separate experiments, each with 6 technical replicates. All attempts at replication (at least twice) were successful.

Randomization

LC-MS and PTR-MS injections were carried out in random order with regular injections of blanks and known concentrations to control drift. There were no animal or patient therapeutic studies which would necessitate randomisation, and therefore formal randomisation was not performed for the other experiments.

Blinding

LC-MS and PTR-MS data were analysed and processed by an author who was blinded to the nature of the sample. Unblinding was performed after the final concentration-per-mg-tissue was calculated. Similarly for immunophenotyping experiments core-scoring was performed by independent pathologists without knowledge of the index patients' clinical features, or independent pathologist scores. Data processing for the ALDH qPCR study was blinded to the sample type. Once the data had been collected and processed to a final form, the data was unblinded (by SA and ZB).

Reporting for specific materials, systems and methods

We require information from authors about some types of materials, experimental systems and methods used in many studies. Here, indicate whether each material, system or method listed is relevant to your study. If you are not sure if a list item applies to your research, read the appropriate section before selecting a response.

Materials & experimental systems

n/a	Involved in the study
☐	☒ Antibodies
☐	☒ Eukaryotic cell lines
☒	☐ Palaeontology and archaeology
☒	☐ Animals and other organisms
☐	☒ Human research participants
☐	☒ Clinical data
☒	☐ Dual use research of concern

Methods

n/a	Involved in the study
☒	☐ ChIP-seq
☒	☐ Flow cytometry
☒	☐ MRI-based neuroimaging

Antibodies

Antibodies used

Antibodies:
ALDH1A3 Abgent AP7847A
ALDH2 Abcam ab108306
ALDH3A1 Sigma HPA051150
ALDH3A2 Sigma HPA014769
ALDH4A1 Abnova 1A12-A5
ALDH9A1 Sigma HPA010873
α-Tubulin Cell Signalling Technologies DM1A
Cyclophilin B Cell Signalling Technologies D1V5J
E-cadherin ThermoFisher NCH38
Keratins CK5/6 Merck D5/16 B4
p63 Abcam 4A4
TP53 Cell Signalling Technologies DO-1
Vimentin Sigma V9
Phospho-Histone H2AX (Ser139) Cell Signalling Technologies 20E3
Phospho-ATM (Ser1981) Cell Signalling Technologies D6H9
Phospho-ATR (Ser428) Cell Signalling Technologies 2853
Phospho-CHK1 (Ser345) Cell Signalling Technologies 133D3
Phospho-CHK2 (Thr68) Cell Signalling Technologies C13C1

Validation

The ALDH3A1, 3A2, 9A1 antibodies (Prestige brand) are rabbit polyclonal antibodies reactive against the human polypeptide and have all been validated by western blotting and immunohistochemistry as specific to the intended target, as detailed in the Human Protein Atlas (https://www.proteinatlas.org/). They were further validated in our lab by western blotting (appropriate size and expected expression as predicted by PCR) and immunohistochemistry (appropriate cell expression and subcellular location).

The ALDH1A3 antibody (https://www.abcepta.com/products/AP7847a-ALDH1A3-Antibody-N-term) is a rabbit polyclonal antibody reactive against the human polypeptide and has been validated by western blotting and immunohistochemistry as specific to the intended target. This antibody was further validated in our lab by western blotting (appropriate size and expected expression as predicted by PCR).

The ALDH2 antibody is a well cited rabbit polyclonal antibody reactive against the human polypeptide (PMID: 32732978, 28729482) and is validated by western blot and immunohistochemistry by the manufacturer (https:// www.abcam.com/), and was further validated in our lab by western blotting (appropriate size and expected expression as predicted by PCR).

The ALDH4A1 antibody is mouse monoclonal antibody reactive against the human polypeptide has been validated by western blot and immunohistochemistry by the manufacturer (http://www.abnova.com/products/products_detail.asp?catalog_id=H00008659-M01). This antibody was further validated in our lab by western blotting (appropriate size and expected expression as predicted by PCR) and immunohistochemistry (appropriate cell expression and subcellular location).

The following antibodies are all mouse monoclonal antibodies recognizing the corresponding human antigen have been validated for immunohistochemistry and immunoblotting use. We internally validated these antibodies in our lab as they recognized specific bands at appropriate sizes by immunoblotting. In addition, all of these antibodies have been validated for use for clinical diagnosis.
E-cadherin Thermofisher NCH38 (https://www.thermofisher.com/antibody/product/E-cadherin-Antibody-clone-NCH-38-Monoclonal/MA5-12547)
Keratins CK5/6 Merck D5/16 B4 (https://www.sigmaaldrich.com/catalog/product/mm/mab1620?lang=en&region=GB)
p63 Abcam 4A4 (https://www.abcam.com/p63-antibody-4a4-ab735.html)
TP53 Cell Signalling Technologies DO-1 (https://www.cellsignal.co.uk/products/primary-antibodies/p53-antibody/9282)
Vimentin Sigma V9 (https://www.sigmaaldrich.com/catalog/product/sigma/347m1?lang=en&region=GB)

The following antibodies have been validated by the manufacturer (https://www.cellsignal.co.uk/) for immunoblotting, and validated internally by our lab by recognizing appropriate and specific bands. All are rabbit polyclonal antibodies recognizing the corresponding human epitope.
Phospho-Histone H2AX (Ser139) Cell Signalling Technologies 20E3
Phospho-ATM (Ser1981) Cell Signalling Technologies D6H9
Phospho-ATR (Ser428) Cell Signalling Technologies 2853
Phospho-CHK1 (Ser345) Cell Signalling Technologies 133D3
Phospho-CHK2 (Thr68) Cell Signalling Technologies C13C1

See manufacturers websites provided for validation statements found in technical data sheets.

Eukaryotic cell lines

Policy information about cell lines

Cell line source(s)	These commercial cell lines were purchased from the ECACC (via Sigma):<br>FLO-1 (Male) Sigma 11012001<br>OACM5.1 (Female) Sigma 11012006<br>ESO26 (Male) Sigma 11012009<br>KYAE-1 (Male) Sigma 11012002<br>OE33 (Female) Sigma 10092313<br><br>These commercial cell lines were purchased from ATCC (via LGC standards)<br>CPA ATCC CRL-4027<br>CPB ATCC CRL-4028<br>CPD ATCC CRL-4030
Authentication	All commercial cell lines were purchased with STR reports from ECACC and ATCC, confirming authenicity
Mycoplasma contamination	All cell lines were negative for mycoplasma. They were tested at the start and end of the study, and at 3 monthly intervals.
Commonly misidentified lines (See ICLAC register)	No commonly misidentified lines were used in this study

Human research participants

Policy information about studies involving human research participants

Population characteristics	Patients were drawn from a tertiary care setting receiving treatment for esophageal adenocarcinoma. The population characteristics of the LC-MS and PTR-MS study cohorts are provided in Supplementary Table 4. In summary, there were no significant differences between the cancer patients and healthy controls in relevant co-variates, including age, gender, smoking or alcohol history, reflux symptoms or proton pump inhibitor use.
Recruitment	Recruitment for the LC-MS study was consecutive based on referrals to our tertiary care multidisciplinary meeting. Three patients refused to participate (patient choice). These occurred at random and were not expected to introduce a selection bias or otherwise impact the overall results. Moreover, this study concluded two years before the Covid-19 pandemic, and thus not influence by the recent changes in stage-at-diagnosis and clinical practice.
Ethics oversight	Fresh human material and associated clinical metadata was collected & accessed under UK National Research Ethics Service Ref: 14/LO/0742, Imperial College Healthcare Tissuebank Approvals R14067, R14087, and R16018. Archived paraffin embedded tissue was accessed under UK National Research Ethics Service Ref: 14/LO/0742, Imperial College Healthcare Tissuebank, approval R14067, and through UK National Research Ethics Service Ref: 10/H0305/1. Written informed consent was taken from all patients.

Note that full information on the approval of the study protocol must also be provided in the manuscript.

Clinical data

Policy information about clinical studies

All manuscripts should comply with the ICMJE guidelines for publication of clinical research and a completed CONSORT checklist must be included with all submissions.

Clinical trial registration	Fresh human material and associated clinical metadata was collected & accessed under UK National Research Ethics Service Ref: 14/LO/0742, Imperial College Healthcare Tissuebank Approvals R14067, R14087, and R16018. Archived paraffin embedded tissue was accessed under UK National Research Ethics Service Ref: 14/LO/0742, Imperial College Healthcare Tissuebank, approval R14067, and through UK National Research Ethics Service Ref: 10/H0305/1. Written informed consent was taken from all patients.
Study protocol	This was a prospective observational study collecting biospecimens and metadata
Data collection	This was a prospective observational study collecting biospecimens. Metadata was collected at the point of sampling, and outcome data was collected at one year. Survival studies were conducted on archived paraffin-embedded tissues, or on samples with at least three years follow-up
Outcomes	The primary outcome was molecular phenotypes. Secondary stratification by clinical metadata was carried out in select experiments.
